# Wildfire smoke-related PM_2.5_ concentration measurements, perceived indoor air quality, and health symptoms among Southern California residents during the 2025 Los Angeles wildfires: A latent class mediation approach

**DOI:** 10.1016/j.envint.2026.110207

**Published:** 2026-03-21

**Authors:** Ryan Lee, Jennifer B Unger, Stefan Schneider, Eli Turovsky, Daniel Soto, Eric Kawaguchi, Fred Lurmann, Nathan Pavlovic, Frank Gilliland

**Affiliations:** a California State University, Northridge, Department of Health Sciences, Northridge, CA, USA; b University of Southern California, Keck School of Medicine, Department of Population and Public Health Sciences, Los Angeles, CA, USA; c University of Southern California, Department of Psychology, Los Angeles, CA, USA; d Sonoma Technology, Inc., Petaluma, CA, USA

**Keywords:** Wildfire smoke, PM_2.5_ exposure, Indoor air quality, Health symptoms, Latent class analysis

## Abstract

**Introduction:**

Numerous studies have linked wildfire exposure to adverse physical and mental health outcomes and symptoms. However, few studies incorporate both outdoor wildfire smoke-related PM_2.5_ concentration and indoor air quality measurements. Understanding the mechanisms by which objectively measured and perceived wildfire smoke impact people’s health could facilitate interventions to mitigate adverse health effects.

**Methods:**

Survey data were obtained from N = 849 adult residents in the Los Angeles area 2–3 months after the 2025 wildfires. A latent class analysis identified subgroups of people with similar symptom experiences. Associations between wildfire smoke-related PM_2.5_ concentration and indoor air quality with likely class membership were examined.

**Results:**

We identified three latent subgroups: Physical and Mental Health Symptoms, Physical Health Symptoms, and Low Symptoms. Higher outdoor wildfire smoke-related PM_2.5_ levels were associated with a higher likelihood of belonging to the symptomatic classes, and indoor air quality statistically explained most of this association.

**Discussion:**

Indoor exposure may be an important mechanism by which people are exposed to wildfire smoke, which can cause adverse health symptoms. While outdoor PM_2.5_ concentration is commonly used in wildfire exposure research, our findings suggest that perceived indoor air quality provides additional explanatory information about who experiences more severe symptom profiles, particularly for wildland-urban interface fires where many residents are sheltering in place. Protective behaviors and interventions to maintain clean indoor air during WUI fire events should be promoted.

## 1. Introduction

### 1.1. The 2025 Los Angeles wildland-urban interface wildfires

The January 2025 Los Angeles (LA) wildland-urban interface (WUI) wildfires, including the Palisades and Eaton Fires, were a major environmental disaster that directly resulted in 31 civilian deaths, caused extensive structural damage, and placed almost 200,000 people under evacuation orders (Los Angeles County Medical Examiner. Los Angeles County Medical Examiner, 2025; Stelloh et al., 2025). In addition to destroying or damaging more than 18,000 buildings, the Palisades and Eaton Fires burned more than 37,000 acres of land and caused an estimated $250 billion in economic losses, reflecting one of the most expensive natural disasters in US history (Chang et al., 2025). It took over three weeks to contain these wildfires, and elevated levels of air pollution persisted in the LA basin for subsequent weeks (Schollaert et al., 2025a,b; Benmarhnia et al., 2025). There were also major health implications resulting from the LA WUI fires, with an estimated additional 440 excess deaths from secondary health effects such as smoke exposure and difficulty accessing healthcare (Paglino et al., 2025). These effects were likely also exacerbated by the release of hazardous pollutants such as heavy metals and other toxins and carcinogens from the burning of structures, vehicles, and other industrial/synthetic materials (Lopez et al., 2023; Schammel et al., 2024; Campos and Abrantes, 2021; Zhu et al., 2024; Holder et al., 2023; Tang et al., 2025).

### 1.2. Health effects of wildfire exposure

Numerous studies have linked wildfire exposure to adverse physical and mental health outcomes and symptoms. Wildfire smoke contains a complex mixture of pollutants, including fine particulate matter (PM_2.5_), carbon monoxide, nitrogen oxides, and ozone (Tang et al., 2025). Smoke released from WUI fires contains additional toxic emissions, including hydrogen cyanide, polycyclic aromatic hydrocarbons (PAHs), dioxins and furans, numerous other volatile organic compounds (ie: benzene toluene, xylenes, styrene, and formaldehyde) and metals (ie: lead, chromium, cadmium, and arsenic) (Holder et al., 2023). These pollutants enter the human body primarily through inhalation, but also through ingestion and dermal absorption, which subsequently enter systemic circulation and trigger oxidative stress and systemic inflammation, mechanisms that are responsible for many of the observed health outcomes (Li et al., 2019; Reid et al., 2016; Xu et al., 2020). Acute respiratory symptoms such as coughing, wheezing, and shortness of breath are common during wildfire events, while chronic exposure has been linked to increased incidence of asthma, chronic obstructive pulmonary disease (COPD), and cardiovascular events including myocardial infarction and stroke (Grant and Runkle, 2022; Kondo et al., 2019; Wettstein et al., 2018).

Wildfires also affect mental health. Populations exposed to wildfire smoke often experience trauma related to evacuation, property loss, and environmental disruption. Studies have documented elevated rates of post-traumatic stress disorder (PTSD), depression, and anxiety following wildfire disasters (Lee et al., 2025; Wah et al., 2025; Lei et al., 2024; Gould et al., 2024). More recently, attention has turned to the neurological consequences of wildfire smoke including impaired cognitive function, fatigue, and neuroinflammation, potentially increasing the risk of neurodegenerative diseases over time (Cohen et al., 2025; Chen et al., 2024; White, 2024; Cleland et al., 2022; Du et al., 2024; Zhang et al., 2023). Although human epidemiological data remain limited, early findings underscore the urgency of further investigation into the brain-health impacts of wildfire smoke.

### 1.3. Challenges in wildfire smoke exposure assessment

One challenge within the wildfire literature is the inconsistent measurement of population-level wildfire exposure. This makes it difficult to accurately assess the health risks associated with wildfire smoke. Many studies utilize ambient PM_2.5_ concentration, collected via ground monitoring, satellite-remote sensing, and chemical transport modeling to estimate population-level exposure (Youssouf et al., 2014; Liu et al., 2015).; Childs et al., 2022; Aguilera et al., 2023; Picciotto et al., 2024). Others assess exposure using “smoke-day” indicators, identifying days when smoke from fire events raises PM_2.5_ levels above defined thresholds (Grant and Runkle, 2022; Lei et al., 2024), or “smoke wave” indicators based on the number or duration of multiday periods of enhanced PM_2.5_ concentrations due to wildfire smoke (Casey et al., 2024; Liu et al., 2017). The metrics employed in these methods also use ambient wildfire smoke as a proxy for individual smoke exposure, typically based on residential history. In contrast, studies focusing on occupational settings have used job-specific exposure histories and lung function assessments to capture acute and cumulative effects among firefighters and related personnel (Wah et al., 2025).

While these approaches illustrate various methods designed to capture the magnitude of wildfire exposures, typically relying on ambient PM_2.5_ concentrations, there are still major gaps in accurately estimating health impacts of individuals’ wildfire exposure, especially in the general population. In particular, few studies reporting on the health effects from wildfire exposure examine indoor air quality in conjunction with outdoor PM_2.5_ concentration, despite the fact that indoor smoke exposure may be a critical determinant of health risk. For example, some evidence suggests that housing characteristics related to smoke infiltration rates, such as heating, ventilation, and cooling (HVAC) systems, can mediate health effects of smoke exposure (Sklar et al., 2024). Although measuring indoor air quality on a population level is challenging, it has important implications for prevention because housing characteristics, such as better insulated homes or HEPA filters, can significantly reduce the amount of wildfire smoke that gets into peoples’ homes (O’Dell et al., 2022; Liang et al., 2021; Shrestha et al., 2019). This issue is particularly salient for WUI fires in urban and suburban areas, where many people may live in close proximity to the fires but do not need to evacuate, as was the case for the 2025 LA wildfires. Therefore, residents may shelter in their homes for the duration of the fires and have disproportionately high levels of exposure, especially when they live in older homes that are poorly insulated or do not have central HVAC systems with HEPA filters (Chan et al., 2013). This highlights some of the inequities and health disparities caused by WUI fires, as lower-income individuals are more likely to live in older homes, not have access to expensive central HVAC systems, and be unable to travel away from smoky conditions (Scott et al., 2025). This is especially concerning given the median age of the dwellings in Los Angeles is 1965, compared to 1977 for California and 1981 across the entire US (US Census Bureau, 2025), and only an estimated 19.6% of homes in Los Angeles have central air conditioning (Ahn and Uejio, 2022), highlighting the degree of vulnerability for wildfire smoke to infiltrate into older homes that do not have HVAC systems.

### 1.4. The present study

In this study, we first conducted a latent class analysis (LCA) of health symptoms experienced by Southern California residents during the 2025 LA WUI fires. To our knowledge, no previous studies have utilized a person-centered approach, such as LCA, to identify subgroups of people with similar wildfire symptom patterns. This approach is useful because it helps identify latent subgroups defined by shared symptom profiles, rather than examining each symptom in isolation or solely focusing on respiratory or cardiovascular symptoms. LCA can also highlight vulnerable subgroups that may benefit from targeted interventions and services during future WUI fires. Similar LCA approaches have been applied in other health contexts, for example, to characterize patterns long COVID symptomatology (Kumar et al., 2025).

Second, we examined predictors of class membership utilizing multinomial logistic regression models. Given that prior research on the health effects of wildfire exposure rarely incorporates both wildfire smoke-related PM_2.5_ exposure and indoor air quality measurements, we conducted a mediation analysis examining both the direct effects of average hourly wildfire smoke-related PM_2.5_ concentration on likely class membership (identified from the LCA) and indirect effects mediated through perceived indoor air quality ratings. We hypothesized that higher wildfire smoke-related PM_2.5_ concentration would be associated with greater likelihood of belonging to symptomatic classes, and that perceived indoor air quality would partially mediate this relationship.

## 2. Methods

### 2.1. Participants

This study included Southern California residents who had participated in a prior study of COVID-19 and mental health in 2021–2022, called the Trojan Pandemic Response Initiative (TPRI) (Lee et al., 2022). The TPRI study consisted of students, staff, and faculty from the University of Southern California (USC).

### 2.2. Procedure

In March-April 2025, 6,889 previous TPRI participants were invited to participate in the current study, Project Firestorm. Participants were eligible if they still lived in Southern California in 2025. Of those invited to participate, 1,141 responded (16.6%), with 893 (78.3%) meeting eligibility criteria and providing written electronic informed consent. The relatively modest response rate is likely due to outdated contact information because the university automatically disables email accounts after students and staff leave; the most recent contact with the TPRI participants was in 2022 via their university email addresses. To assess potential nonresponse bias, we tested for differences in demographic characteristics between respondents and non-respondents; respondents were more likely to be Female/Other gender, older age, and White or Hispanic compared to non-respondents (Table S1). Participants received a $10 gift card for their participation. The University of Southern California Institutional Review Board approved this study (UP-25–00077).

### 2.3. Measures

#### 2.3.1. Health symptoms

Health symptoms from the wildfires were assessed by asking respondents, “Did you experience any of the following symptoms while breathing in smoke in January 2025?” with a checklist of 16 different health symptoms (See Table 1 for the list of symptoms). Respondents had the option to select all that applied.

#### 2.3.2. Wildfire smoke-related PM_2.5_ exposure

We constructed and geocoded residential timelines for the primary wildfire period (January 7–12) for each participant based on residential addresses (and evacuation addresses where applicable). Occupancy dates and times were obtained for each address the participant resided in during the fire period. Geocodes were provided with a date of evacuation that included a time of day reported by the participant as “Morning”, “Afternoon”, or “Evening”. To assign smoke-related PM_2.5_ as described below, the periods beginning at each time of day were assigned using hourly values starting at 00:00 (Morning), 12:00 (Afternoon), or 19:00 (Evening). Similarly, periods ending at each time of day were assigned using hourly values up to 11:00 (Morning), 18:00 (Afternoon), or 23:00 (Evening). Overlapping hourly data at the origin and destination locations were averaged before assignments were made, accounting for uncertainty of the exact timing of the evacuation. Several geocodes provided a date without a time of day. In these cases, the start hour was set to 00:00 and the end hour was set to 23:00.

To estimate wildfire smoke PM_2.5_ exposure at high spatiotemporal resolution for each study participant, we used wildfire smoke PM_2.5_ dispersion model results with observed hourly PM_2.5_ from regulatory monitors and low-cost sensors. Hybrid Single-Particle Lagrangian Integrated Trajectory (HYSPLIT) dispersion modeling at 1 km resolution for January 7 through 12 was used to estimate wildfire smoke-related PM_2.5_ (Stein et al., 2015; Li et al., 2020; Kim et al., 2020). Smoke-related PM_2.5_ emissions were calculated from satellite-measured fire radiative power (FRP) from the GOES Fire Detection and Characterization (FDC) algorithm applied to imagery collected by the Advanced Baseline Imagers (ABI) onboard the East and West Geostationary Operational Environmental Satellites (GOES-16 and GOES-18) (Schroeder et al., 2010; Schmit et al., 2017). Emissions were calculated from GOES hourly mean FRP using the Fire Energetics and Emissions Research (FEER) v1.0 co-efficient of emissions product (Ichoku and Ellison, 2014). Dispersion was run using 3 km resolution High Resolution Rapid Refresh (HRRR) meteorology. To ensure consistency with observed PM_2.5_, HYSPLIT concentrations were scaled to match the range of quality assured observations of hourly total PM_2.5_. Observations were obtained from regulatory monitors in U.S. Environmental Protection Agency (EPA) AirNow and from low-cost sensors from the Clarity Node-S and PurpleAir networks.The PurpleAir data were calibrated using the Barkjohn et al 2022 correction equation (Barkjohn et al., 2022) and the Clarity Node-S data were calibrated and quality assured using the Version 2 Global Calibration Model for PM_2.5_ described in the Supplementary material. All observation data were independently reviewed for quality. Scaling was performed based on HYPSLIT concentrations extracted at all observation locations. For each day, a scaling factor was calculated across all observations based on hourly data as maximum of the ratios of the observations to HYPSLIT. This scaling ensured that concentrations did not exceed the maximum of observations at monitoring sites while allowing higher concentration estimates away from observations. The daily scaling factor was applied to all smoke PM_2.5_ values from HYSPLIT for each day.

**Fig. 1.**
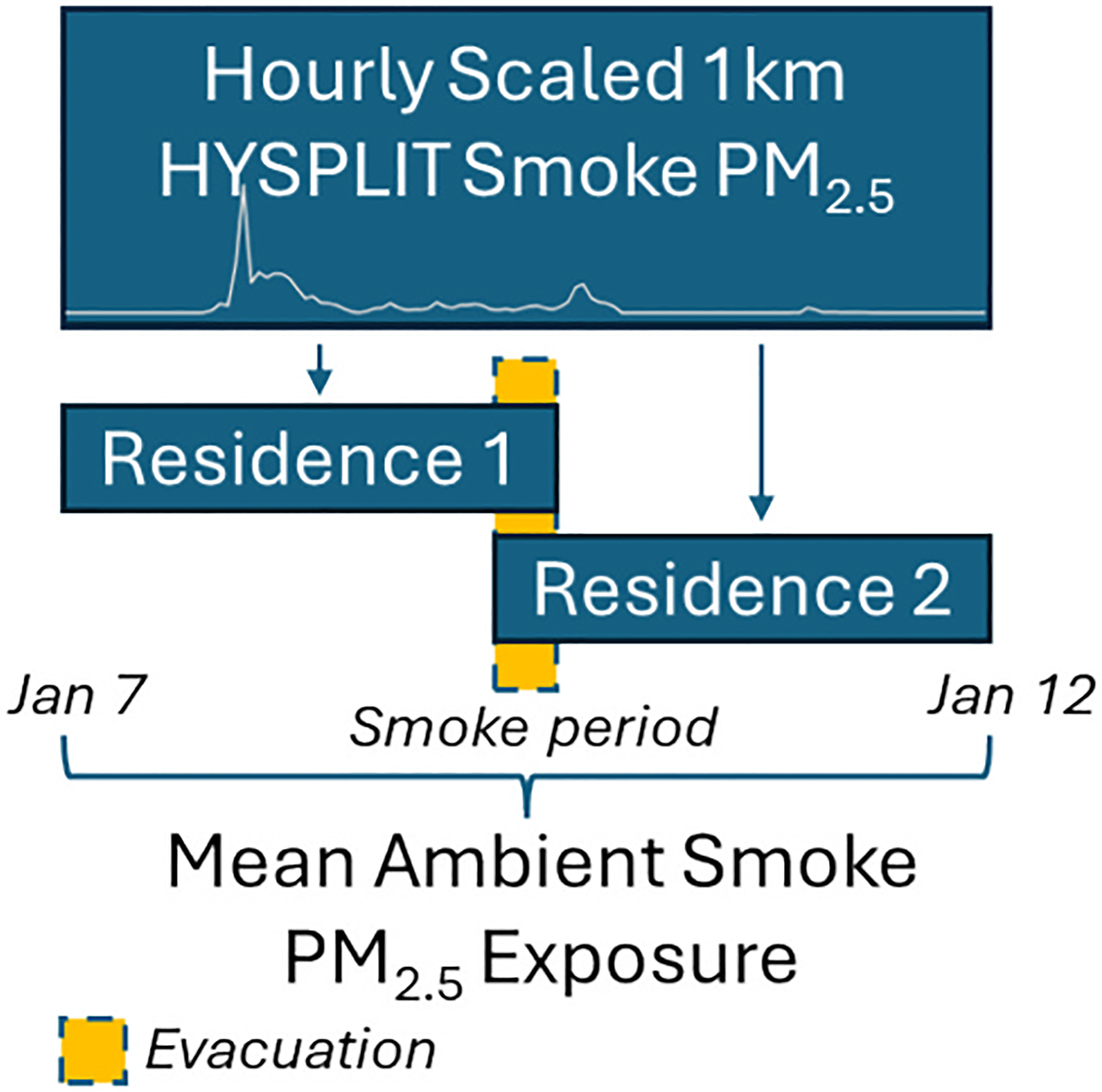
Ambient smoke PM_2.5_ exposure assignment method. Gray line indicates the mean hourly smoke PM_2.5_ exposure across all study participants for the smoke period from January 7 to January 12. Values range from 0 to 190 μg/m^3^ PM_2.5_.

Hourly smoke-related PM_2.5_ was assigned for each participant during the fire period, and the mean wildfire smoke-related PM_2.5_ exposure across the fire period was calculated (Fig. 1). The continuous mean wildfire smoke-related PM_2.5_ variable was standardized (Mean = 0, SD = 1) to improve interpretability such that a one-unit increase reflects a one standard deviation increase in PM_2.5_ exposure.

#### 2.3.3. Perceived indoor air quality rating

Perceived indoor air quality was assessed using a single-item self-report measure from the survey asking participants, “How would you describe the indoor air quality during the wildfire?” with the following response options: 1 = Very good (no noticeable smoke), 2 = Somewhat smoky (mild odor/haze), 3 = Moderately smoky (noticeable odor/haze), 4 = Very smoky (strong odor, visible haze), 5 = Unsure. For this study, the “Unsure” response option (3.2% of respondents) was re-coded to “1 – Very good” and a sensitivity analysis (Table S2) was conducted in which the “Unsure” response option was recoded as missing and the key study results remained stable. In this study, perceived indoor air quality was treated as a continuous variable, however, a sensitivity analysis where indoor air quality was modeled as categorical is included in the Supplemental materials and does not change the main results (Table S3).

#### 2.3.4. Hours outside

The amount of time spent outside during the wildfires was assessed using a single-item self-report measure from the survey asking participants, “During the worst smoke days, how many hours per day were you outdoors?” with the following response options: 1 = Less than 1 h, 2 = 1–2 h, 3 = 3–4 h, 4 = More than 4 h.

#### 2.3.5. Baseline health status

Baseline health status was assessed using a single-item self-report measure from the survey asking participants, “How would you rate your overall health before the wildfire?” with response options ranging from 1 = Excellent to 4 = Poor.

#### 2.3.6. Demographics

Self-reported race/ethnicity, gender, and age were assessed in previous TPRI surveys. Due to small group sizes, African American/Black, Multi-racial/ethnic, and “Other” race/ethnicity were combined into one “Other” category, resulting in four race/ethnicity categories: Non-Hispanic White, Asian, Hispanic, and Other. Similarly, Transgender, Non-binary, and “Other” gender identities were combined with Female gender due to small group sizes.

### 2.4. Data analysis

First, a LCA was performed to identify subgroups of individuals (latent classes) with similar symptom experiences during the wildfires (Vermunt and Magidson, 2002; Collins and Lanza, 2009). Mplus Version 8 (Muthén and Muthén, 2017) was used to estimate the LCA with a Full Information Maximum Likelihood estimator, which is the default estimator in Mplus.

To identify the appropriate number of classes, we compared several model fit statistics, including Akaike information criterion (AIC), Bayesian information criterion (BIC), sample-size adjusted Bayesian Information Criterion (saBIC) (Sclove, 1987), and entropy. Model selection was based on the following criteria: 1) lower AIC, BIC and saBIC values, indicating better model fit; 2) higher entropy values, indicating better class separation and homogeneity (Collins and Lanza, 2009; Ferguson et al., 2020); 3) class sizes, with greater proportions of the sample in each class; and 4) parsimonious classes with theoretically meaningful subgroups (Nylund-Gibson and Choi, 2018; Spurk et al.,2020).

After selecting the final number of latent classes, we examined how wildfire exposure variables, baseline health status, and demographic variables were associated with class membership. These associations were estimated using multinomial regression analysis within the LCA framework, implemented in Mplus with the maximum likelihood 3-step approach (Asparouhov and Muthén, 2014; Vermunt, 2010). In the first step, we estimated the unconditional LCA model without covariates. In the second step, each participant’s most likely class membership and the associated classification probabilities were saved. The third step involves regressing the most-likely-class-membership variable onto the covariates whilst incorporating classification uncertainty. We used Mplus’s automated R3STEP implementation of this procedure, which allows multiple covariates to be examined simultaneously and provides regression estimates adjusted for all other covariates in the model. It is the recommended approach for studies examining the effect of observed predictors on latent classes (Asparouhov and Muthén, 2021).

Finally, to investigate whether indoor air quality mediated the association between outdoor wildfire smoke-related PM_2.5_ and class membership, we then specified a mediation model using the same three-step ML framework but implemented manually following Bakk, Tekle, and Vermunt (2013). As in the covariate model above, the unconditional LCA was first estimated, modal class membership and logits for classification probabilities were saved, and in the third step the measurement parameters were fixed while specifying the auxiliary structural model linking wildfire smoke-related PM_2.5_ concentration, perceived indoor air quality, and class membership. This approach accounts for classification uncertainty and has been shown to perform well when latent classes serve as outcome variables in mediation models (Hsiao et al., 2021).

## 3. Results

Of the 893 participants enrolled in the study, 44 did not reach the health symptoms section of the survey, resulting in an analytical sample of N = 849. Demographic characteristics of the study sample are reported in Table 1. The sample was majority female/other gender (71.4%), Non-Hispanic White (34.8%) and Asian (31.2%), and had a mean age of 35.5 years. Smoke period mean smoke-related PM_2.5_ exposures ranged from 0 to 149 μg/m^3^, with a median exposure of 8 μg/m^3^. The most commonly reported health symptoms during the wildfires were scratchy throat (41.7%), dry cough (35.1%), stinging eyes (31.3%), headache (30.5%), and anxiety (29.8%).

To identify the optimal number of latent classes in the LCA, we examined model fit indices for two to four classes (Table 2). In the two, three, and four class solutions, the AIC and saBIC decreased with each additional class, indicating improved model fit with each additional class solution; however, the BIC increased between the 3-class and 4-class solutions, suggesting the 3-class solution had better fit than the 4-class solution. The entropy values were >0.7 in each class solution. Based on the combination of these model fit indices, sample size of each class, and the interpretability of the identified classes, the three-class model was identified as the optimal model.

**Fig. 2.**
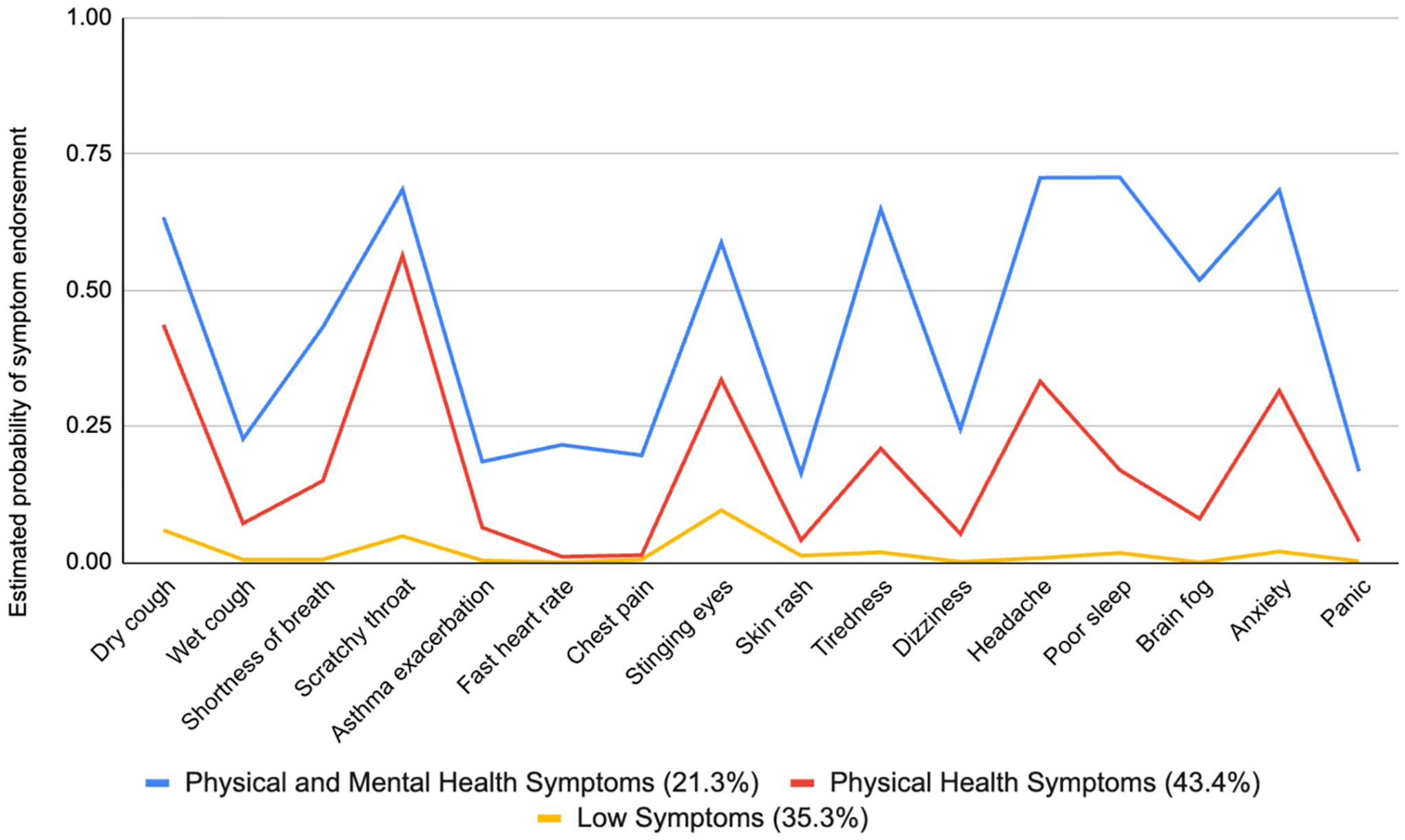
Estimated probability for endorsing health symptoms during the wildfires by class membership (N = 849).

Results from the LCA are shown in Fig. 2. The first class, labeled “Low Symptoms” (with 35.3% of the sample expected to belong to this class), included participants who reported little to no symptoms. The second class, labeled “Physical Health Symptoms” (with 43.4% of the sample expected to belong to this class) included participants who reported primarily physical health symptoms during the fires, namely dry cough, scratch throat, stinging eyes, and headache, though anxiety was also reported in this group. The third class, labeled “Physical and Mental Health Symptoms” (with 21.3% of the sample expected to belong to this class) included participants who reported similar physical symptoms as the “Physical Health Symptoms” class, but also additional mental and neurological health symptoms such as tiredness, poor sleep and brain fog in addition to anxiety. This class also had the highest estimated probability of endorsing each symptom compared to the other two classes (see Fig. 2). For example, dry cough, starchy throat, tiredness, headache, poor sleep, and anxiety were endorsed by 60–75% of the members in the Physical and Mental Health Symptoms class.

Bivariate multinomial logistic regression analyses (Table 3) indicated that higher wildfire smoke-related PM_2.5_ concentration was associated with greater odds of belonging to the Physical and Mental Health Symptoms group (OR = 1.04, p = 0.03) and Physical Health Symptoms group (OR = 1.04, p = 0.01), compared to the Low Symptoms group. However, PM_2.5_ concentration did not distinguish between the two symptomatic groups (OR = 0.99, p = 0.60).

Poorer perceived indoor air quality was associated with greater odds of belonging to the Physical and Mental Health Symptoms (OR = 4.76, p < 0.001) and Physical Health Symptoms (OR = 2.24, p = 0.001) groups compared to the Low Symptoms group. It also increased the likelihood of belonging to the Physical and Mental Health Symptoms group rather than the Physical Symptoms only group (OR = 2.12, p = 0.001).

Next we examined an adjusted model examining the association between PM_2.5_ concentration and likely class membership controlling for perceived indoor air quality and other key covariates (baseline heath status, the amount of time spent outdoors during the fires, age, gender, and race/ethnicity) (Table 4). In this adjusted model, the previously significant bivariate associations between PM_2.5_ concentration and membership in the symptomatic classes (vs. Low Symptoms) became nonsignificant (AOR = 1.00, p = 0.89; AOR = 1.03, p = 0.08, respectively). In contrast, perceived indoor air quality remained a significant predictor of likely class membership after controlling for PM_2.5_ concentration and other covariates. Poorer perceived indoor air quality was associated with higher odds of membership in the Physical and Mental Health Symptoms (AOR = 4.32, p < 0.001) and Physical Health Symptoms (AOR = 1.92, p = 0.009) compared to the Low Symptoms group. Perceived indoor air quality also remained significantly associated with likely Physical and Mental Health Symptoms group membership compared to Physical Symptoms only (AOR = 2.25, p = 0.002).

**Fig. 3.**
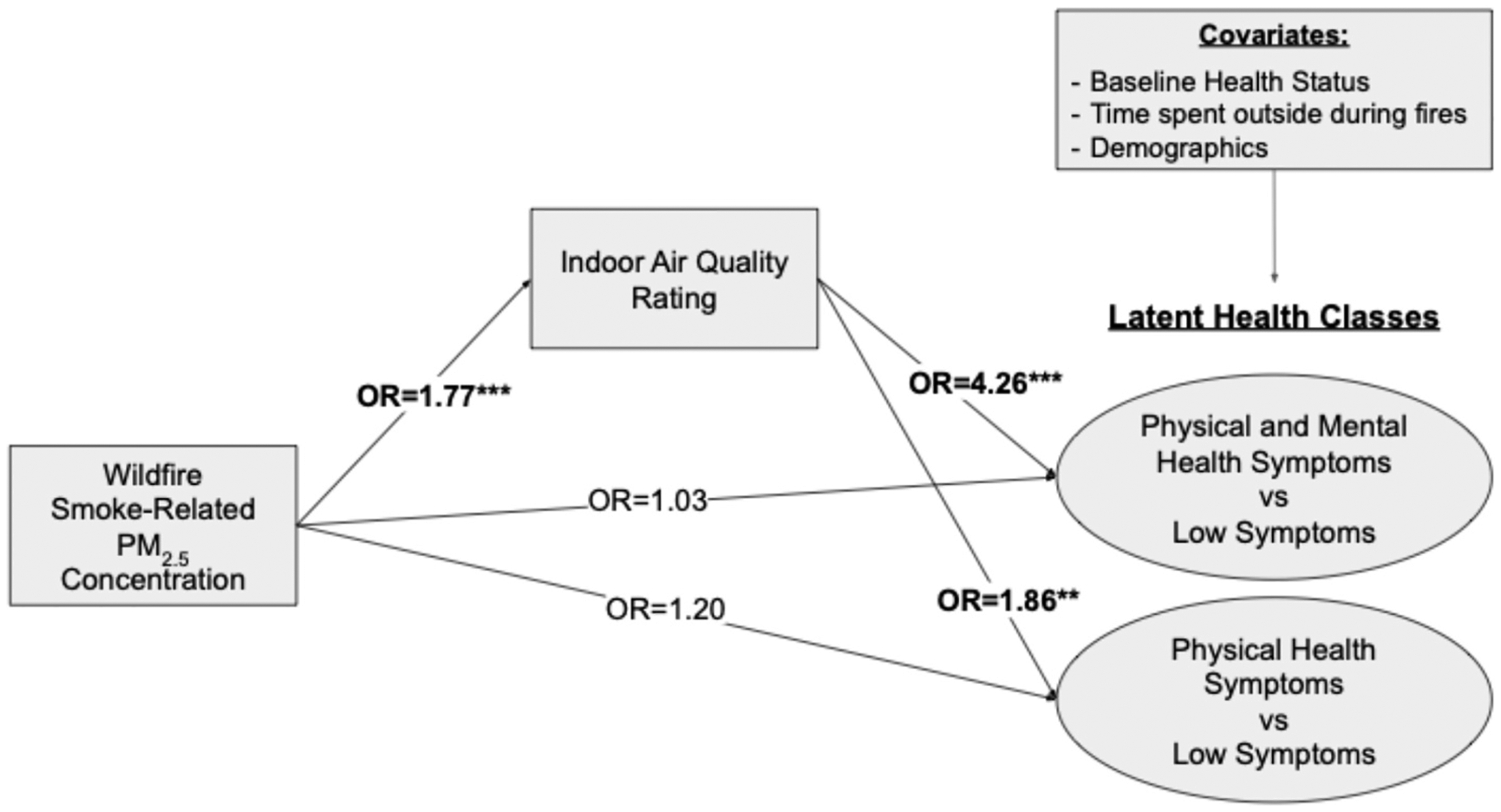
Mediation model examining direct and indirect effects of wildfire smoke-related PM2.5 concentration on likely latent health class assignment via indoor air quality rating. The indirect effect of wildfire smoke-related PM_2.5_ concentration → indoor air quality → Physical & Mental Health Symptoms Class vs Low Symptoms Class was OR_Indirect_ = 2.28, p < 0.001, Proportion Mediated effect = 96.2%. The indirect effect of wildfire smoke-related PM_2.5_ concentration → indoor air quality → Physical Health Symptoms Class vs Low Symptoms Class was OR_Indirect_ = 1.42, p < 0.001, Proportion Mediated effect = 66.2%. (N = 849).

In the mediation model (Fig. 3), wildfire smoke-related PM_2.5_ concentration was positively associated with perceived indoor air quality rating (OR = 1.77, p < 0.001), but was not directly associated with membership in either the Physical and Mental Health Symptoms class nor the Physical Health Symptoms class versus the Low Symptoms class (OR = 1.03, p = 0.82; OR = 1.20, p = 0.22, respectively). In contrast, perceived indoor air quality was strongly positively associated with membership in both the Physical and Mental Health Symptoms and the Physical Health Symptoms classes versus the Low Symptoms class (OR = 4.26, p < 0.001; OR = 1.86, p = 0.004, respectively). The indirect effect of wildfire smoke-related PM_2.5_ concentration on Physical & Mental Health Symptoms Class (vs Low Symptoms Class), mediated through perceived indoor air quality, was significant (OR_Indirect_ = 2.28, p < 0.001, proportion mediated effect = 96.2%). A similar indirect effect was observed for the Physical Health Symptoms Class (vs Low Symptoms Class) (OR_Indirect_ = 1.42, p < 0.001, proportion mediated effect =66.2%).

## 4. Discussion

Wildfires are becoming an increasingly common and severe natural disaster, with both acute and long-term health implications for those exposed. These wildfires are also increasingly occurring in urban and suburban areas (WUI fires) where many people may live in close proximity to the fires but do not evacuate, resulting in large numbers of residents sheltering in their homes and being exposed to high levels of wildfire smoke. WUI fires are of particular concern because the smoke can contain not only particulate matter from burnt organic material, but also heavy metals and other toxins and carcinogens released from the burning of structures, vehicles, and other industrial/synthetic materials (Lopez et al., 2023; Schammel et al., 2024; Campos and Abrantes, 2021; Zhu et al., 2024). While the health effects of wildfire exposure have been well studied (Wah et al., 2025; Lei et al., 2024; Gould et al., 2024; Reid et al., 2016), few studies investigated WUI fires with as much structural damage as the 2025 LA wildfires, and, to our knowledge, no studies have conducted a LCA to identify subgroups of people experiencing similar health symptoms.

Utilizing a LCA approach, we identified three latent classes of health symptoms experienced by Southern California residents impacted by the January 2025 LA wildfires: (1) Low Symptoms, (2) Physical Health Symptoms, and (3) Physical and Mental Health Symptoms. These findings highlight the heterogeneity in health symptoms experienced by Southern California residents as a result of WUI fire exposure and underscore the mental and neurological health implications wildfire exposure can have in addition to commonly reported respiratory and cardiovascular symptoms. Consistent with previous research, our study identified common wildfire-related respiratory and mental health symptoms, such as scratchy throat, dry cough, stinging eyes, and anxiety (Wah et al., 2025; Lei et al., 2024). Our study also identified various wildfire-related neurological health symptoms, including headache, tiredness, poor sleep and brain fog. These results are consistent with emerging research on the negative impact of wildfire smoke on brain and neurological health (Cohen et al., 2025; Chen et al., 2024); however, to our knowledge, brain fog as a health symptom has not yet been documented in the wildfire health literature. Similar symptoms to brain fog such as cognitive impairment have been reported among populations exposed to wildfire smoke (Grennan et al., 2023; White, 2025; Du et al., 2024). However, with brain fog only becoming widely studied during and after the COVID-19 pandemic due to it being a common symptom of long COVID (Haywood et al., 2025; Kumar et al., 2025), it is likely that brain fog was underrecognized in previous studies on wildfire health impacts. Now that better diagnostic and screening measures for brain fog exist, researchers should study this condition further to understand the risks that wildfire smoke may have on brain fog (Debowska et al., 2024).

In addition to identifying latent subgroups of fire-related health symptomology, we also examined the associations of two exposure measures (wildfire smoke-related PM_2.5_ concentration and self-rated indoor air quality) with likely membership in these latent subgroups. Despite the existing literature on the health effects from wildfire smoke exposure, few studies have measured wildfire exposure on a population level using both measures of PM_2.5_ concentration and indoor air quality. Our goal was to explore whether higher levels of exposure increased one’s odds of belonging to a worse health symptomology group and to test whether indoor air quality functioned as the mechanism linking wildfire smoke-related PM_2.5_ concentration to wildfire health symptomology. Our results demonstrated perceived indoor air quality accounted for most of the relationship between wildfire smoke-related PM_2.5_ concentration on belonging to latent classes characterized by having worse symptoms. Additionally, when controlling for perceived indoor air quality rating in the same model, the significant bivariate effects of outdoor smoke exposure become non-significant. These results suggest indoor smoke exposure may be an important mechanism by which people are exposed to wildfire smoke, which can cause adverse health symptoms.

While PM_2.5_ is commonly examined in the wildfire literature, our findings suggest it might not be sufficient to measure personal exposure to outdoor wildfire smoke, particularly for WUI fires where many residents are sheltering in place. This underscores indoor air quality, not only as an important source of wildfire smoke exposure, but also as an important variable for researchers to measure and account for in their studies on the health effects of wildfires. As wildfires continue to encroach on urban and suburban areas, this will likely exacerbate existing health disparities as residents living in older or poorly insulated housing, or those without HVAC systems with HEPA filters, will have higher indoor smoke levels. Simultaneously, many low-income residents, who are more likely to live in these types of homes, may be unable to voluntarily evacuate their homes during times of wildfire smoke due to the financial burden of evacuation-related costs. Future studies on the health impacts of wildfires should include indoor air quality measurements whenever feasible because basing findings on outdoor measurements alone could underestimate actual exposures and misclassify health hazards. Additionally, protective behaviors to maintain clean indoor air during WUI fire events should be promoted, such as keeping windows closed, using AC/heat systems on recirculate, and using HEPA filters/air purifiers when possible (Chen et al., 2026). Disaster management officials may also want to consider expanding the area for voluntary evacuation for residents living near future WUI fires, despite not being in direct danger from the fires themselves, due to the risk of indoor smoke exposure on adverse health effects for those sheltering in place.

## 5. Limitations

Due to the rapid initiation of this study following the wildfires, this study employed convenience sampling leveraging a previous cohort of university affiliated students, staff, and faculty who were still living in the greater Los Angeles area during the 2025 LA wildfires. Therefore, findings may not generalize to non-university-affiliated populations, including children, older adults, or low-SES populations. Nevertheless, because wildfire smoke exposure was widespread across the greater Los Angeles area, university affiliation is unlikely to have meaningfully biased participants’ exposure levels or the health symptoms they reported during the fires. Additionally, participants in this study resided across a large geographic area and did not only concentrate around the USC campuses (see distribution of study sample residence in Lee et al., 2025).

Another limitation in this study is the moderate sample size and cross-sectional nature of the study. While this is an important limitation to recognize, establishing a large representative cohort of Southern California residents in the immediate aftermath of a wildfire poses significant challenges, making alternative sampling strategies a necessary consideration. For this reason, the study team decided to re-recruit an existing cohort of Southern California residents that were able to be rapidly contacted post-fire. However, these participants had not been contacted in over two years, and it is likely that many had their university email address deactivated upon graduating or leaving USC. This led to a low response rate to our study invitation. Comparison of our study sample to the original TPRI sample (Table S1) demonstrated differences in the demographic makeup between the two samples, suggesting our results may be impacted by non-response bias.

Lastly, our survey measures were all self-report, including measures of indoor air quality and health symptoms experienced during the fires. The cross-sectional nature of the survey subjects the study to recall bias and limits our ability to establish temporal ordering or causality. For example, the directionality of the relationship between health symptoms and perceived air quality has the potential to be bi-directional. While we controlled for demographics and baseline health to approximate sequential ignorability, we did not employ a formal counterfactual mediation framework. Current implementations of these causal effects for latent class outcomes in Mplus utilize a logistic link function, which requires a ‘rare outcome assumption’ (prevalence < 10%) to avoid biased estimates (McLarnon and O’Neill, 2018). Because our symptom classes were prevalent (21%–43%), this assumption was violated, and we instead utilized the 3-step associative approach to ensure latent class stability. Additionally, indoor air quality was a one-item survey question that was assessed 2–3 months after the LA wildfires. Thus, this measure may be subject to recall bias. Additionally, the one-item measure had limited variation due to only five response options, including an “unsure” option and it is also possible that perceived indoor air quality ratings may have been inflated by visual cues of intense outdoor smoke during the fire-period. Ideally, future studies should incorporate objective measures of indoor air quality to validate our study findings. However, recognizing indoor air quality monitors are uncommon in most households, future studies can utilize better survey measures and real-time data capture, such as Ecological Momentary Assessment (EMA) to improve variation, accuracy, temporality, and reduce recall bias.

## 6. Conclusion

This study underscores the importance of indoor air quality in understanding the health hazards related to wildfire exposure. This study also documents a potential novel wildfire-related neurological health symptom, brain fog. As wildfires intensify and become increasingly prevalent, especially in densely populated WUI communities, public health research and interventions should also incorporate measures to record and reduce indoor smoke exposure. Protective behaviors to maintain clean indoor air during WUI fire events should be promoted, such as keeping windows closed, using AC/heat systems on recirculate, and using HEPA filters/air purifiers when possible. Further research is needed to confirm and explore the mechanisms underlying wildfire-induced brain fog and to determine interventions that can reduce the physical as well as cognitive burden of wildfire smoke exposure.

## Supplementary Material

1

## Figures and Tables

**Table 1:** Descriptive statistics of study sample.

	N(%)	Mean(SD)
**Age**		35.5 (14.2)
**Gender**		
Male	239 (28.6%)	
Female/Other	597 (71.4%)	
**Race**		
White	284 (34.8%)	
Asian	255 (31.2%)	
Hispanic	151 (18.5%)	
Other	127 (15.5%)	
**Wildfire smoke-related PM**_**2.5**_ **concentration**^a^		8.7 (8.2)
**Indoor Air Quality**		
Very good (no noticeable smoke)	329 (39.3%)	
Somewhat smoky (mild odor/haze)	313 (37.4%)	
Moderately/Very smoky (noticeable or strong odor, visible haze)	195 (23.3%)	
**Time Spent Outdoors During Wildfires**		
Less than 1 h	588 (70.1%)	
1–2 h	191 (22.8%)	
3 or more hours	60 (7.2%)	
**Baseline Health**		
Excellent	318 (37.9%)	
Good	451 (53.7%)	
Fair/Poor	71 (8.5%)	
**Health Symptoms**		
Dry cough	292 (35.1%)	
Wet cough	69 (8.3%)	
Shortness of breath	135 (16.2%)	
Scratchy throat	347 (41.7%)	
Asthma exacerbation	58 (7.0%)	
Fast heart rate	44 (5.3%)	
Chest pain	42 (5.1%)	
Stinging eyes	260 (31.3%)	
Skin rash	49 (5.9%)	
Tiredness	203 (24.4%)	
Dizziness	65 (7.8%)	
Headache	254 (30.5%)	
Poor sleep	199 (23.9%)	
Brain fog	124 (14.9%)	
Anxiety	248 (29.8%)	
Panic	46 (5.5%)	

a Wildfire smoke-related PM_2.5_ concentration was measured as hourly average ranging from 0 – 105.7 μg/m3

**Table 2:** Model fit indices of Latent Class Analysis.

Classes	AIC	BIC	saBIC	Entropy
2	10178.78	10334.99	10230.19	0.811
3	10058.56	10295.23	10136.44	0.717
4	9989.26	10306.40	10093.63	0.733

* AIC = Akaike Information Criterion, BIC = Bayesian information criterion, saBIC = Sample Size Adjusted BIC; N = 840.

**Table 3:** Bivariate multinomial logistic regression results examining associations between wildfire smoke-related PM_2.5_ concentration, indoor air quality, and other demographic characteristics with likely class membership (N = 849).

	Physical and Mental Health Symptoms vs. Low Symptoms		Physical Health Symptoms vs. Low Symptoms		Physical and Mental Health Symptoms vs. Physical Symptoms	
	OR	p-value	OR	p-value	OR	p-value
**Wildfire smoke-related PM**_**2.5**_ **concentration**	**1.036**	**0.027**	**1.042**	**0.014**	0.994	0.600
**Indoor Air Quality**	**4.755**	**<0.001**	**2.241**	**0.001**	**2.121**	**0.001**
**Hours outside**	**1.570**	**0.034**	1.189	0.360	1.321	0.190
**Baseline Health**	**2.264**	**0.002**	1.455	0.072	1.556	0.059
**Age**	0.996	0.591	0.999	0.885	0.997	0.706
**Gender**						
Male	*ref*	*ref*	*ref*	*ref*	*ref*	*ref*
Female/Other	**3.468**	**0.011**	**1.786**	**0.041**	1.941	0.123
**Race**						
White	*ref*	*ref*	*ref*	*ref*	*ref*	*ref*
Asian	1.007	0.981	0.882	0.584	1.141	0.699
Hispanic	**3.491**	**0.028**	1.918	0.144	1.820	0.171
Other	1.381	0.375	1.381	0.375	1.090	0.827

* Note, bold indicates significance at p<0.05.

**Table 4:** Multivariable multinomial logistic regression results examining associations between wildfire smoke-related PM_2.5_ concentration, indoor air quality, and other demographic characteristics with likely class membership (N = 849).

	Physical and Mental Health Symptoms vs. Low Symptoms		Physical Health Symptoms vs. Low Symptoms		Physical and Mental Health Symptoms vs. Physical Symptoms	
	AOR	p-value	AOR	p-value	AOR	p-value
**Wildfire smoke-related PM**_**2.5**_ **concentration**	1.003	0.888	1.03	0.075	0.973	0.075
**Indoor Air Quality**	**4.324**	**<0.001**	**1.924**	**0.009**	**2.247**	**0.002**
**Hours outside**	1.459	0.141	1.209	0.377	1.207	0.407
**Baseline Health**	**2.065**	**0.022**	1.369	0.161	1.508	0.136
**Age**	0.999	0.91	1.003	0.709	0.996	0.688
**Gender**						
Male	*ref*	*ref*	*ref*	*ref*	*ref*	*ref*
Female/Other	**3.071**	**0.032**	1.728	0.084	1.778	0.181
**Race**						
White	*ref*	*ref*	*ref*	*ref*	*ref*	*ref*
Asian	0.762	0.37	0.742	0.203	1.026	0.946
Hispanic	1.846	0.299	1.629	0.337	1.133	0.757
Other	0.925	0.838	1.135	0.725	0.815	0.575

* Note, bold indicates significance at p<0.05.

## Data Availability

Data will be made available on request.

## Appendix A. Supplementary data

Supplementary data to this article can be found online at https://doi.org/10.1016/j.envint.2026.110207.
