# Supplementary material for: Wildfire smoke-related PM2.5 concentration measurements, perceived indoor air quality, and health symptoms among Southern California residents during the 2025 Los Angeles wildfires: A latent class mediation approach
Source: Environ Int. Author manuscript; Available in PMC 2026 Jun 1. (PMC13224745; doi:10.1016/j.envint.2026.110207)
Supplement: 1 [file NIHMS2179593-supplement-1.docx]

**Supplemental Materials**

**Table S1.** Comparison of demographics of respondents (vs. non-respondents) from the original TPRI sample invited to participate in the current study.

|  | **Non-Respondents (N=6049)** | **Respondents**  **(N=849)** | **Group difference**  **test** |
| --- | --- | --- | --- |
|  | N(%) or u(SD) | N(%) or u(SD) | p-value |
| **Gender** |  |  | <0.001 |
| Man | 2122 (36.3%) | 239 (28.6%) |  |
| Woman/Other | 3724 (63.7%) | 597 (71.4%) |  |
| **Race/ethnicity** |  |  | 0.001 |
| White | 1774 (30.5%) | 284 (34.8%) |  |
| Asian | 2044 (35.2%) | 255 (31.2%) |  |
| Hispanic | 892 (15.4%) | 151 (18.5%) |  |
| Other | 1102 (19.0%) | 127 (15.5%) |  |
| **Age** | u=29.7 (SD=12.2) | u=35.3 (SD=14.0) | <0.001 |

*Note: Independent t-test for continuous variables and χ2 test for categorical variables were conducted.*

**Table S2.** Multivariable multinomial logistic regression results - where indoor air quality “unsure” response option is re-coded as missing (N=849)

|  | **Physical and Mental Health Symptoms**  **vs**  **Low Symptoms** | | **Physical Health Symptoms**  **vs**  **Low Symptoms** | | **Physical and Mental Health Symptoms**  **vs**  **Physical Symptoms** | |
| --- | --- | --- | --- | --- | --- | --- |
|  | **AOR** | **p-value** | **AOR** | **p-value** | **AOR** | **p-value** |
| **Wildfire smoke-related PM_2.5_ concentration** | 0.999 | 0.942 | 1.025 | 0.14 | 0.974 | 0.086 |
| **Indoor Air Quality** | **4.339** | **<0.001** | **1.923** | **0.01** | **2.256** | **0.003** |
| **Hours outside** | 1.443 | 0.152 | 1.23 | 0.345 | 1.173 | 0.472 |
| **Baseline Health** | **2.045** | **0.023** | 1.348 | 0.191 | 1.517 | 0.126 |
| **Age** | 0.999 | 0.896 | 1.003 | 0.72 | 0.996 | 0.685 |
| **Gender** |  |  |  |  |  |  |
| Male | *ref* | *ref* | *ref* | *ref* | *ref* | *ref* |
| Female | **3.069** | **0.033** | 1.82 | 0.071 | 1.686 | 0.218 |
| **Race** |  |  |  |  |  |  |
| White | *ref* | *ref* | *ref* | *ref* | *ref* | *ref* |
| Asian | 0.75 | 0.345 | 0.752 | 0.234 | 0.997 | 0.993 |
| Hispanic | 1.807 | 0.312 | 1.626 | 0.342 | 1.111 | 0.791 |
| Other | 0.95 | 0.896 | 1.181 | 0.66 | 0.805 | 0.551 |

**Table S3.** Multivariable multinomial logistic regression results - where indoor air quality is modeled as categorical rather than continuous (N=849)

|  | **Physical and Mental Health Symptoms**  **vs**  **Low Symptoms** | | **Physical Health Symptoms**  **vs**  **Low Symptoms** | | **Physical and Mental Health Symptoms**  **vs**  **Physical Symptoms** | |
| --- | --- | --- | --- | --- | --- | --- |
|  | **AOR** | **p-value** | **AOR** | **p-value** | **AOR** | **p-value** |
| **Wildfire smoke-related PM_2.5_ concentration** | 1.001 | 0.945 | 1.029 | 0.085 | 0.973 | 0.074 |
| **Indoor Air Quality** |  |  |  |  |  |  |
| Good | *ref* | *ref* | *ref* | *ref* | *ref* | *ref* |
| Mild | **4.635** | **0.016** | **3.007** | **0.01** | 1.542 | 0.311 |
| Moderate/Poor | **13.66** | **0.014** | 2.603 | 0.091 | **5.248** | **0.035** |
| **Hours outside** | 1.435 | 0.149 | 1.17 | 0.466 | 1.227 | 0.379 |
| **Baseline Health** | **2.038** | **0.023** | 1.358 | 0.177 | 1.5 | 0.146 |
| **Age** | 0.999 | 0.952 | 1.004 | 0.617 | 0.995 | 0.647 |
| **Gender** |  |  |  |  |  |  |
| Male | *ref* | *ref* | *ref* | *ref* | *ref* | *ref* |
| Female | **2.959** | **0.032** | 1.656 | 0.114 | 1.787 | 0.178 |
| **Race** |  |  |  |  |  |  |
| White | *ref* | *ref* | *ref* | *ref* | *ref* | *ref* |
| Asian | 0.741 | 0.312 | 0.705 | 0.141 | 1.051 | 0.9 |
| Hispanic | 1.728 | 0.331 | 1.495 | 0.408 | 1.156 | 0.728 |
| Other | 0.921 | 0.828 | 1.135 | 0.731 | 0.812 | 0.566 |

**Table S4.** Multivariable logistic regression models examining PM_2.5_ concentration and perceived indoor air quality on each health symptom individually, controlling for age, gender, race, baseline health status, hours outside during fires.

| **Outcome (% endorsed)** | **Predictor** | **AOR** | **p-value** | **LR Test** |
| --- | --- | --- | --- | --- |
| Dry cough (35%) | **PM_2.5_** | 1.031 | 0.711 |  |
|  | **Indoor Air Quality** |  |  | **<0.001** |
|  | Very good | *ref* | *ref* |  |
|  | Somewhat smoky | **2.064** | **<0.001** |  |
|  | Moderately/Very smoky | **2.768** | **<0.001** |  |
| Wet cough (8%) | **PM_2.5_** | 0.863 | 0.352 |  |
|  | **Indoor Air Quality** |  |  | **0.0195** |
|  | Very good | *ref* | *ref* |  |
|  | Somewhat smoky | 1.433 | 0.322 |  |
|  | Moderately/Very smoky | **2.735** | **0.007** |  |
| Shortness of breath (16%) | **PM_2.5_** | 1 | 0.999 |  |
|  | **Indoor Air Quality** |  |  | **0.0005** |
|  | Very good | *ref* | *ref* |  |
|  | Somewhat smoky | **2.007** | **0.01** |  |
|  | Moderately/Very smoky | **2.948** | **<0.001** |  |
| Scratchy throat (41%) | **PM_2.5_** | 1.051 | 0.554 |  |
|  | **Indoor Air Quality** |  |  | **<0.001** |
|  | Very good | *ref* | *ref* |  |
|  | Somewhat smoky | **2.008** | **<0.001** |  |
|  | Moderately/Very smoky | **2.993** | **<0.001** |  |
| Asthma exacerbation (7%) | **PM_2.5_** | 0.879 | 0.425 |  |
|  | **Indoor Air Quality** |  |  | 0.0898 |
|  | Very good | *ref* | *ref* |  |
|  | Somewhat smoky | 2.153 | 0.055 |  |
|  | Moderately/Very smoky | 2.258 | 0.059 |  |
| Fast or irregular heart rate (5%) | **PM_2.5_** | 0.851 | 0.408 |  |
|  | **Indoor Air Quality** |  |  | **0.0006** |
|  | Very good | *ref* | *ref* |  |
|  | Somewhat smoky | 1.634 | 0.334 |  |
|  | Moderately/Very smoky | **5.085** | **0.001** |  |
| Pain or tightness in the chest (5%) | **PM_2.5_** | 0.873 | 0.463 |  |
|  | **Indoor Air Quality** |  |  | **0.0113** |
|  | Very good | *ref* | *ref* |  |
|  | Somewhat smoky | **2.756** | **0.043** |  |
|  | Moderately/Very smoky | **4.189** | **0.005** |  |
| Stinging, itchy, or watery eyes (31%) | **PM_2.5_** | 1.037 | 0.678 |  |
|  | **Indoor Air Quality** |  |  | **<0.001** |
|  | Very good | *ref* | *ref* |  |
|  | Somewhat smoky | **1.747** | **0.007** |  |
|  | Moderately/Very smoky | **3.357** | **<0.001** |  |
| Skin rash (5%) | **PM_2.5_** | 0.798 | 0.258 |  |
|  | **Indoor Air Quality** |  |  | **0.011** |
|  | Very good | *ref* | *ref* |  |
|  | Somewhat smoky | 1.6 | 0.322 |  |
|  | Moderately/Very smoky | **3.692** | **0.005** |  |
| Tiredness (24%) | **PM_2.5_** | 1.089 | 0.351 |  |
|  | **Indoor Air Quality** |  |  | **<0.001** |
|  | Very good | *ref* | *ref* |  |
|  | Somewhat smoky | 1.504 | 0.069 |  |
|  | Moderately/Very smoky | **2.87** | **<0.001** |  |
| Dizziness (8%) | **PM_2.5_** | 1.005 | 0.972 |  |
|  | **Indoor Air Quality** |  |  | **0.0058** |
|  | Very good | *ref* | *ref* |  |
|  | Somewhat smoky | 1.706 | 0.192 |  |
|  | Moderately/Very smoky | **3.461** | **0.002** |  |
| Headache (30%) | **PM_2.5_** | 1.089 | 0.339 |  |
|  | **Indoor Air Quality** |  |  | **<0.001** |
|  | Very good | *ref* | *ref* |  |
|  | Somewhat smoky | **2.031** | **0.001** |  |
|  | Moderately/Very smoky | **3.447** | **<0.001** |  |
| Poor sleep (23%) | **PM_2.5_** | 0.955 | 0.631 |  |
|  | **Indoor Air Quality** |  |  | **<0.001** |
|  | Very good | *ref* | *ref* |  |
|  | Somewhat smoky | **1.875** | **0.007** |  |
|  | Moderately/Very smoky | **3.468** | **<0.001** |  |
| Brain fog (15%) | **PM_2.5_** | 0.9 | 0.376 |  |
|  | **Indoor Air Quality** |  |  | **0.0005** |
|  | Very good | *ref* | *ref* |  |
|  | Somewhat smoky | 1.667 | 0.066 |  |
|  | Moderately/Very smoky | **3.052** | **<0.001** |  |
| Anxiety (30%) | **PM_2.5_** | 1.05 | 0.576 |  |
|  | **Indoor Air Quality** |  |  | **<0.001** |
|  | Very good | *ref* | *ref* |  |
|  | Somewhat smoky | **1.559** | **0.033** |  |
|  | Moderately/Very smoky | **3.368** | **<0.001** |  |
| Panic (5%) | **PM_2.5_** | 0.783 | 0.242 |  |
|  | **Indoor Air Quality** |  |  | 0.2104 |
|  | Very good | *ref* | *ref* |  |
|  | Somewhat smoky | 1.534 | 0.341 |  |
|  | Moderately/Very smoky | 2.289 | 0.08 |  |

Clarity Node-S LCS Quality Assurance

Clarity automatically applies a quality assurance check to data to determine cases where the uncalibrated PM_1_ concentration is unreasonably high compared to the uncalibrated PM_2.5_ concentration. Data were removed if ratio of PM_1_ to PM_2.5_ is above a ratio limit that decreases linearly with increasing PM_1_ concentration over a set of concentration ranges according to the table below.

| PM_1_ Concentration (μg/m3) | Ratio limit |
| --- | --- |
| <= 55 | None |
| 55-75 | 1.0-0.9 |
| 75-175 | 0.9-0.8 |
| 175-1200 | 0.8-0.5 |
| >=1200 | 0.5 (Fixed) |

Clarity Node-S LCS V2 Calibration equation

The version 2 calibration equation applied to Clarity Node-S used in this work is calculated as:

v2 Calibrated PM2.5 =

(Raw PM_2.5_ concentration) * 0.274821 +

(Raw PM_10_ concentration) * 0.263883 +

(Raw PM_1_ concentration) * 0.171146 +

(Relative Humidity) * -0.073857 +

pm_rh_interaction * -0.004631 +

temperature_minus_dew * -0.149043 +

8.076738

Where pm_rh_interaction is calculated as Raw PM_2.5_ concentration * (Relative Humidity) and

temperature_minus_dew is calculated as Temperature – Dew Point. The dew point is calculated from relative humidity and temperature using the Magnus formula.

**MPLUS Code:**

TITLE: LCA - Firestorm – Step 1

DATA:

File is "Firestorm_LCA";

VARIABLE:

NAMES ARE

pid

drycough

wetcough

shortbreath

scratchthroat

asthma

heartrate

chestpain

stingeyes

skinrash

tiredness

dizziness

headache

poorsleep

brainfog

anxiety

panic

z_smoke_pm

indoor_air

hrs_outside

baseline_health

asian

hispanic

other

age

gender2

;

CATEGORICAL ARE

drycough

wetcough

shortbreath

scratchthroat

asthma

heartrate

chestpain

stingeyes

skinrash

tiredness

dizziness

headache

poorsleep

brainfog

anxiety

panic

;

USEVARIABLES ARE

drycough

wetcough

shortbreath

scratchthroat

asthma

heartrate

chestpain

stingeyes

skinrash

tiredness

dizziness

headache

poorsleep

brainfog

anxiety

panic

;

MISSING ARE ALL(-999);

CLASSES = c(3);

AUXILIARY =

z_smoke_pm

indoor_air

hrs_outside

baseline_health

asian

hispanic

other

age

gender2

;

ANALYSIS:

TYPE = MIXTURE;

STARTS = 500 50;

STITERATIONS = 50;

PROCESSORS = 4;

MODEL:

%OVERALL%

SAVEDATA:

FILE = "vam_weights.dat";

SAVE = CPROB;

FORMAT IS FREE;

MISSFLAG=-999;

PLOT:

type=plot2;

series =

drycough (1)

wetcough (2)

shortbreath (3)

scratchthroat (4)

asthma (5)

heartrate (6)

chestpain (7)

stingeyes (8)

skinrash (9)

tiredness (10)

dizziness (11)

headache (12)

poorsleep (13)

brainfog (14)

anxiety (15)

panic (16)

;

OUTPUT:

tech11 tech14;

TITLE: LCA - Firestorm – Step 2

DATA:

File is "vam_weights.dat";

VARIABLE:

NAMES ARE

DRYCOUGH

WETCOUGH

SHORTBRE

SCRATCHT

ASTHMA

HEARTRAT

CHESTPAI

STINGEYE

SKINRASH

TIREDNES

DIZZINES

HEADACHE

POORSLEE

BRAINFOG

ANXIETY

PANIC

Z_SMOKE_

INDOOR_A

HRS_OUTS

BASELINE

ASIAN

HISPANIC

OTHER

AGE

GENDER2

CPROB1

CPROB2

CPROB3

MODAL

;

USEVARIABLES ARE

Z_SMOKE_

INDOOR_A

HRS_OUTS

BASELINE

ASIAN

HISPANIC

OTHER

AGE

GENDER2

MODAL

;

NOMINAL = MODAL;

CATEGORICAL = INDOOR_A;

MISSING ARE ALL(-999);

CLASSES = c(3);

ANALYSIS:

TYPE = MIXTURE;

STARTS=0;

PROCESSORS=4;

ESTIMATOR=ML;

ALGORITHM=INTEGRATION;

INTEGRATION=MONTE;

MODEL:

%OVERALL%

INDOOR_A ON

Z_SMOKE_ (a) ;

Z_SMOKE_;

HRS_OUTS;

BASELINE;

ASIAN;

HISPANIC;

OTHER;

AGE;

GENDER2;

Z_SMOKE_ HRS_OUTS BASELINE ASIAN HISPANIC OTHER AGE GENDER2 WITH

Z_SMOKE_ HRS_OUTS BASELINE ASIAN HISPANIC OTHER AGE GENDER2;

C#1 ON

INDOOR_A (b1) ! path 'b' for Class 1

Z_SMOKE_ (c1) ! direct effect c' for Class 1

HRS_OUTS

BASELINE

ASIAN

HISPANIC

OTHER

AGE

GENDER2;

C#2 ON

INDOOR_A (b2) ! path 'b' for Class 2

Z_SMOKE_ (c2) ! direct effect c' for Class 2

HRS_OUTS

BASELINE

ASIAN

HISPANIC

OTHER

AGE

GENDER2;

! Fix logits for "Classification Probabilities for the Most Likely Latent Class Membership"

%C#1%

[MODAL#1@2.391]; ! row 1, col 1

[MODAL#2@-0.443]; ! row 1, col 2

%C#2%

[MODAL#1@6.502]; ! row 2, col 1

[MODAL#2@8.199]; ! row 2, col 2

%C#3%

[MODAL#1@-2.200]; ! row 3, col 1

[MODAL#2@-13.710]; ! row 3, col 2

MODEL CONSTRAINT:

NEW(ind_C1 ind_C2); ! indirect effect (a*b) in log odds metric

NEW(total_C1 total_C2); !total effect in log odds metric

NEW(prop_C1 prop_c2); !proportion mediated

NEW(OR_ind_C1 OR_ind_C2); ! indirect effect in OR metric

NEW(OR_total_C1 OR_total_C2); ! total effect in OR metric

NEW(OR_a); ! a-path in OR metric

NEW(OR_b1 OR_b2); ! b-paths in OR metric

NEW(OR_c1 OR_c2); ! c-paths in OR metric

ind_C1 = a*b1;

ind_C2 = a*b2;

total_C1 = (a * b1) + c1;

total_C2 = (a * b2) + c2;

prop_C1 = ind_C1 / total_C1;

prop_C2 = ind_C2 / total_C2;

OR_ind_C1 = exp(ind_C1);

OR_ind_C2 = exp(ind_C2);

OR_total_C1 = exp(total_C1);

OR_total_C2 = exp(total_C2);

OR_a = exp(a);

OR_b1 = exp(b1);

OR_b2 = exp(b2);

OR_c1 = exp(c1);

OR_c2 = exp(c2);

MODEL TEST:

ind_C1 = 0;

ind_C2 = 0;
